# Usefulness of *C. elegans* Models of Alzheimer’s and Huntington’s Disease to Evaluate Novel Imidazoline I2 Receptor Ligands

**DOI:** 10.3390/ijms27073282

**Published:** 2026-04-04

**Authors:** Teresa Taboada-Jara, Marta Ribalta, Fernando Romero-Becerra, Joel Muixí, Aina Bellver-Sanchis, Christian Griñán-Ferré, Carmen Escolano, Mercè Pallàs

**Affiliations:** 1 Pharmacology Section, Department of Pharmacology, Toxicology and Medicinal Chemistry, Faculty of Pharmacy and Food Sciences, and Institut de Neurociències (UBNeuro), University of Barcelona, 08028 Barcelona, Spain; 2 Laboratory of Medicinal Chemistry, Department of Pharmacology, Toxicology and Medicinal Chemistry, Faculty of Pharmacy and Food Sciences, University of Barcelona, Av. Joan XXIII, 27–31, 08028 Barcelona, Spain; 3 Institute of Biomedicine (IBUB), University of Barcelona, 08028 Barcelona, Spain; 4 CiberNed—Network Center for Neurodegenerative Diseases, National Spanish Health Institute Carlos III, 28220 Madrid, Spain

**Keywords:** *C. elegans* models, imidazoline I2 receptor ligands, neurodegeneration, Alzheimer’s disease, Huntington’s disease, Aβ aggregation, Ht513 aggregates, oxidative stress, *sod-1* gene expression

## Abstract

Neurodegenerative diseases such as Alzheimer’s (AD) and Huntington’s (HD) remain major therapeutic challenges due to limited treatment efficacy. Imidazoline I2 receptor (I2-IR) ligands have recently emerged as promising neuroprotective agents, with reported roles in modulating oxidative stress, neuroinflammation, and protein aggregation. This study evaluates the therapeutic potential of several I2-IR ligands, including Idazoxan, CR4056, and novel compounds, using *Caenorhabditis elegans* (*C. elegans*) models of AD and HD. Transgenic strains CL2006 (expressing human Aβ1-42) and EAK103 (expressing Ht513) were employed to assess locomotor activity, oxidative stress tolerance, Aβ and Ht aggregation, and *sod-1* gene expression. Several ligands significantly improved movement, reduced Aβ and Ht aggregates, and enhanced antioxidant gene expression, particularly Idazoxan, LSL42, and PIP01. Notably, some compounds exhibited prooxidant effects, highlighting the utility of *C. elegans* for early in vivo toxicity screening. Importantly, this study provides the first in vivo evidence of the efficacy of I2-IR ligands in HD models and reinforces their potential as therapeutic candidates for HD. Overall, these findings suggest a potential role for modulation of I2-IR-related pathways in neurodegeneration and support the utility of *C. elegans* as a rapid, cost-effective platform for preclinical drug evaluation.

## 1. Introduction

Neurodegenerative disorders, particularly Alzheimer’s disease (AD) and Huntington’s disease (HD), which are characterized by progressive neuronal dysfunction and death, represent significant challenges in modern medicine [1,2,3,4,5]. Traditional symptomatic treatments for AD, such as cholinesterase inhibitors (donepezil, galantamine, and rivastigmine) and the NMDA receptor antagonist memantine, remain widely used and provide symptomatic relief and functional stabilization across mild to severe stages of the disease. In 2025, the therapeutic landscape expanded to include anti-amyloid monoclonal antibodies such as lecanemab and donanemab, which showed slow cognitive decline in patients with early-stage AD by clearing amyloid plaques from the brain [6,7]. Over the past two decades, drug development efforts have mainly targeted aggregated proteins central to AD pathology, such as amyloid-beta (Aβ) and tau; however, these approaches have shown limited success, with clinical trials achieving only about a 2% success rate and most agents offering only modest benefits in terms of symptom relief and slowing disease progression [8]. Furthermore, therapies for HD have also been poorly developed, and apart from riluzole, which is authorized in several countries, no disease-modifying therapies are currently available. Therefore, the procurement of novel therapies that effectively slow or halt the progression of AD or HD is crucial for patients.

Imidazoline receptors (IRs), also known as imidazoline binding sites, comprise a heterogeneous family of entities with high affinity for compounds containing an imidazoline moiety. Originally identified as non-adrenergic binding sites, IRs have emerged as key modulators of diverse cellular functions [9,10]. The imidazoline receptor subtype 2 (I2-IR) has garnered significant attention due to its high density in brain regions affected by neurodegenerative processes and its involvement in mitochondrial function and cellular stress responses [11,12,13]. In fact, recent advances in molecular neuroscience have further underscored the potential role of I2-IR modulation in neuroprotection and cellular homeostasis, opening new avenues for therapeutic intervention [14,15,16]. Of note, accumulating evidence suggests that I2-IR ligands may influence protein aggregation and cellular survival pathways implicated in the pathogenesis of both AD and HD [17,18,19].

Imidazoline heterocyclic compounds such as 2-BFI, BU224, and its counterpart imidazole LSL60101, and structurally novel ligands such as MCR5, BIN02, BIN05, B06, and PIP01, characterized by α-phosphonate substituted nitrogen heterocycles, have demonstrated promising pharmacological (see affinity for human I2-IR and selectivity versus αadrenergic receptors in Table A1) and therapeutic effects. Specifically, these compounds have been shown to improve cognitive performance and attenuate neuroinflammation in transgenic mouse models of AD and in senescence-accelerated mouse prone 8 (SAMP8), a widely used model of late-onset AD [20,21,22,23,24,25,26].

Concretely, the first I_2_-IR ligands that exerted a neuroprotective role in a murine model of AD were MCR5 and MCR9 [21,27]. Treatment with compounds from the MCR family led to significant behavioral and cognitive improvements, which were associated with the modulation of several molecular pathways, including oxidative stress (OS), inflammation, synaptic plasticity, apoptosis pathways, amyloid precursor protein processing, and increasing Aβ-degrading enzymes in the hippocampus [21]. Moreover, MCR5 attenuated depressive-like and fear–anxiety-like behaviors, while improving cognitive performance in SAMP8 mice. These effects were linked to reduced neuroinflammation and enhanced synaptic plasticity [27]. Beneficial effects were replicated by other chemically diverse I2-IR ligands, such as B06, LSL60101, or PIP01, in SAMP8 mice or in the 5XFAD transgenic model, a well-established early-onset AD [20,25,26]. Particularly, chronic low-dose treatment with LSL60101 reversed cognitive deficits and impaired social behavior similarly to donepezil. LSL60101 attenuated amyloid-β pathology, reduced microglial markers and glial fibrillary acidic protein (GFAP), and increased Trem2 gene expression, indicating a clear prevention of glial reactivity following I2-IR ligand administration [20].

Moreover, recent studies have demonstrated that bicyclic α-phosphoproline derivatives, including BIN05 and B06-red, exhibited high affinity for I2-IR in human brain tissues (Table A1) and favorable BBB permeation capabilities, exerted neuroprotective activity in neurodegenerative disease models of *C. elegans*. Using a thrashing assay as a functional readout, treatment with BIN05 and B06-red resulted in significant improvement in locomotor performance, indicative of significant cognitive improvement in this in vivo model [22].

*C. elegans* has emerged as a powerful model organism for investigating neurological disorders and accelerating early-stage drug discovery. Its fully mapped nervous system, extensive genetic conservation with humans, optical transparency, and short life cycle make it particularly well suited for dissecting molecular mechanisms underlying neurodegeneration and for conducting high-throughput pharmacological screening. As emphasized by Romussi [28], *C. elegans* models enable efficient evaluation of neuronal function, protein aggregation, lifespan effects, and behavioral phenotypes across broad neurodevelopmental and neurodegenerative conditions [29,30,31]. While the organism’s simplicity limits its ability to capture certain human-specific physiological features, findings obtained in *C. elegans* provide a rapid and cost-effective foundation for subsequent validation in mammalian systems, reinforcing its role as a complementary and highly informative platform in preclinical drug discovery. The ability of *C. elegans* to model key aspects of both AD and HD provides an excellent platform to investigate the therapeutic potential of imidazoline I_2_ receptor modulation, particularly in relation to autophagy [32,33].

Therefore, we aimed to determine the effectiveness of different families of I2-IR ligands in AD, assessing automated movement assay and thioflavin staining using the transgenic strain CL2006, which expresses human Aβ1-42 under control of a muscle-specific promoter and responds to Aβ1-42 aggregation with progressive adult-onset paralysis. Of note, the selection of CL2006 was based on its robust expression of disease-relevant pathology—muscle-localized Aβ aggregates that can be directly quantified by thioflavin-S staining, progressive paralysis as a functional outcome measure, and compatibility with oxidative stress assays. This multi-parametric approach enabled comprehensive evaluation of the neuroprotective mechanisms of I_2_-IR ligands within a single, cost-effective in vivo system. Furthermore, we evaluate OS using WT strain (Bristol N2) and the effect on huntingtin 513 (Htt-513) aggregates in EAK103 strain, a *C. elegans* model of HD (Htt-513) aggregation and toxicity, which occurs in 513 amino acid caspase cleavage, associated with motor dysfunction [34]. Likewise, EAK103 was specifically chosen over other polyQ models because it expresses the pathologically relevant 513-amino acid huntingtin fragment with an 128Q expansion, which recapitulates aggregate formation and motor deficits observed in HD patients. The availability of the EAK102 control strain (15Q) provided internal validation of aggregate-specific therapeutic effects. Given the novelty of this approach, well-established I2-IR ligands, preclinical Idazoxan, and clinical CR4056, with reported neuroprotective properties, were included as reference compounds and evaluated alongside early-stage I2-IR ligands in the *C. elegans* models [35,36].

By integrating behavioral, protein aggregation, biochemical, and molecular analyses in *C. elegans* models using established I2-IR ligands, this study provides a comprehensive framework demonstrating that *C. elegans* is a powerful in vivo system for rapid phenotypic screening of novel I2-IR compounds and other drug candidates targeting neurodegenerative disease pathways. Our findings underscore the value of *C. elegans* as a practical and cost-effective model for elucidating the therapeutic potential of I2-IR modulation in two of the most devastating neurodegenerative diseases, facilitating mechanistic insights that are often challenging to achieve in mammalian models. These findings strengthen the growing body of literature supporting the development of I2-IR-focused therapeutics and highlight *C. elegans* as a reliable and versatile platform for drug discovery in neurodegeneration research.

## 2. Results

### 2.1. In Vivo Toxicity Evaluation of I2-IR Ligands in C. elegans

**Figure 1:**
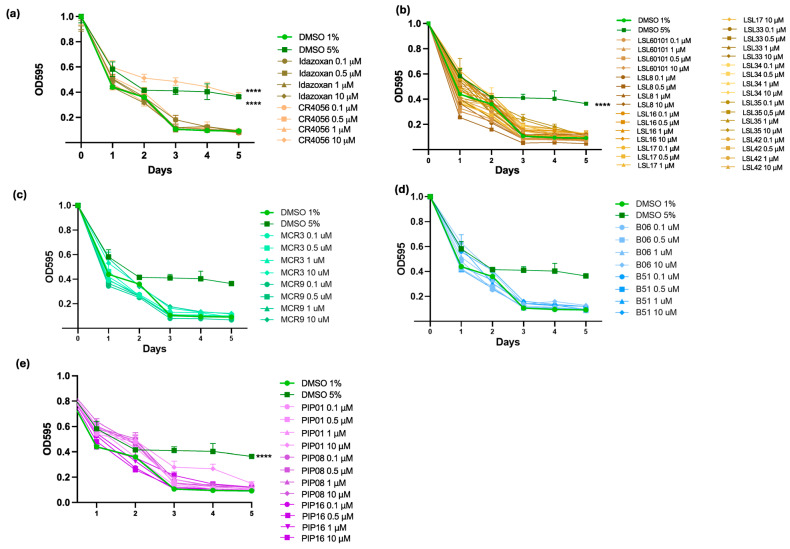
(**a**) Idazoxan and CR4056; (**b**) LSL60101, LSL8, LSL16, LSL17, LSL33, LSL34, LSL35, and LSL42; (**c**) MCR3 and MCR9; (**d**) B06 and B51; and (**e**) PIP01, PIP08, and PIP16 for the food clearance assay. Data are presented as mean ± SEM; *n* = 3, with 120–150 worms per group for each experiment. The data correspond to the mean ± SEM; *n* = 3 with 120–150 worms in each group for each experiment. One-Way ANOVA; **** *p* < 0.001 vs. CL2006. Experimental design and figure organization: All compounds within each panel were tested in parallel across the same three independent biological replicates using identical 1% DMSO vehicle controls. Data are grouped by chemical family to improve visual clarity and support structure–activity relationship analysis. Because all worms received the same standardized vehicle treatment within each synchronized experiment, control values are consistent across panels, ensuring comparability and clear interpretation of related compounds.

Before testing the neuroprotective effect of I2-IR ligands, compound toxicity was assessed in the *C. elegans* wild-type N2 Bristol strain. Figure 1a displays the optical density (OD) of the *Escherichia coli* (*E. coli*) OP50 suspension over the five-day duration of the food clearance assay, which serves as a measure of the worms’ survival. A 1% DMSO solution was used as the vehicle control (negative control), while 5% DMSO served as the positive (toxic) control. The tested compounds at different doses (0.1, 0.5, 1, and 10 μM) were considered safe, as their OD reduction followed a pattern similar to that of the vehicle control, and visual inspection confirmed normal worm development (Figure 1a–d). Only the treatment with 10 μM of CR4056 showed an OD decrease mirroring that of the toxic control (Figure 1b). Finally, a limitation is that toxicity was assessed only in N2 (20 °C), while the disease models (CL2006, EAK103) were maintained at 16 °C. However, no unexpected toxicity was observed in transgenic strains during functional assays at working concentrations (0.1–1 μM).

### 2.2. I2-IR Ligands Increase Movement in CL2006, a C. elegans Model of AD

**Figure 2:**
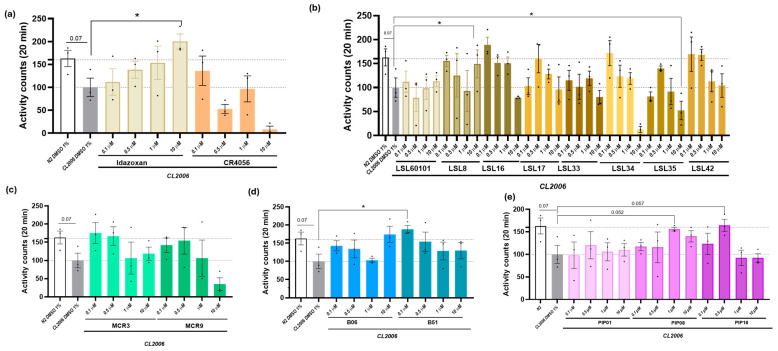
Results from automated movement assay in *C. elegans* (Bristol N2 and CL2006) treated with (0.1, 0.5, 1, and 10 μM) of (**a**) Idazoxan and CR4056; (**b**) LSL60101, LSL8, LSL16, LSL17, LSL33, LSL34, LSL35 and LSL42; (**c**) MCR3 and MCR9; (**d**) B06 and B51; (**e**) PIP01, PIP08 and PIP16 for 4 days. The data correspond to the mean ± SEM; *n* = 3 with 120–150 worms in each group for each experiment. One-Way ANOVA; * *p* < 0.05 vs. CL2006. Experimental design and figure organization: All compounds within each panel were tested in parallel across the same three independent biological replicates using identical 1% DMSO vehicle controls. Data are grouped by chemical family to improve visual clarity and support structure–activity relationship analysis. Because all worms received the same standardized vehicle treatment within each synchronized experiment, control values are consistent across panels, ensuring comparability and clear interpretation of related compounds.

One of the most extensively used transgenic *C. elegans* models of AD is the CL2006 strain, which carries the Punc-54:Aβ1–42 transgene and exhibits a progressive adult-onset paralysis [37]. To establish a dose–response profile, the pharmacological effects of I2-IR ligands were evaluated by measuring the locomotor activity in this strain after each treatment. Both wild-type N2 and CL2006 worms were treated with 1% DMSO, used as a vehicle control, which does not induce significant locomotor defects. As expected, N2 (wild-type) animals showed a higher percentage of swimming movements compared to CL2006 worms, due to the age-dependent paralysis characteristic of the transgenic model. This confirmed that locomotor performance differs between the two strains, enabling assessment of I2-IR ligand effects following chronic exposure (Figure 2a). Some of the I2-IR ligands tested, including Idazoxan (1 μM), increased swimming movements (Figure 2a–e).

### 2.3. I2-IR Ligands Attenuate Oxidative Stress in C. elegans

**Figure 3:**
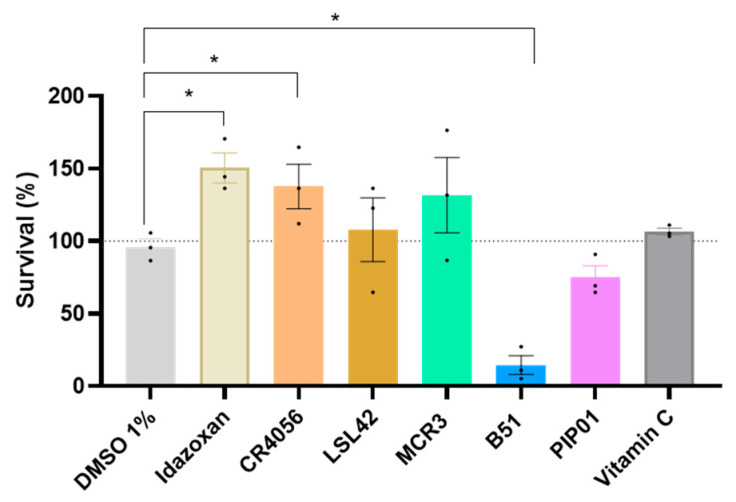
Results from the OS assay in *C. elegans* (Bristol N2) treated with 0.5 μM of Idazoxan, CR4056, LSL42, MCR3, B51, and PIP01 for 4 days after 24 h of exposure to t-butyl hydroperoxide, respectively. Values represented are mean ± standard error of the mean (SEM); *n* = 3, with 120–150 worms in each group for each experiment. One-Way ANOVA; * *p* < 0.05 vs. Vehicle.

To assess whether I2-IR ligands confer protection against OS, the *C. elegans* N2 strain was exposed to the chemical oxidant *tert*-butyl hydroperoxide (6.2 mM) after 4 days of treatment with the tested compounds (0.5 μM). The vitamin C group (58 μM) served as a positive control for antioxidant activity and showed a baseline level of protection against oxidative damage (Figure 3). Several I2-IR ligands significantly enhanced worm survival compared to vehicle control (Figure 3). Notably, Idazoxan and CR4056 conferred robust protection against *tert*-butyl hydroperoxide-induced OS, with survival rates exceeding those observed in the Vitamin C-treated group (Figure 3). Whereas LSL42 and MCR3 showed no significant neuroprotective effect after 24 h of insult. Of note, other I2-IR ligands (B51) potentiate the effect of *tert*-butyl hydroperoxide (Table A2), showing a prooxidant role rather than neuroprotective against this deleterious stimulus, allowing it to add a red flag in the screening process of selection, one of the objectives to use nematodes as a screening in vivo model for drug development.

### 2.4. I2-IR Ligands Inhibit Aβ-Aggregation in CL2006

**Figure 4:**
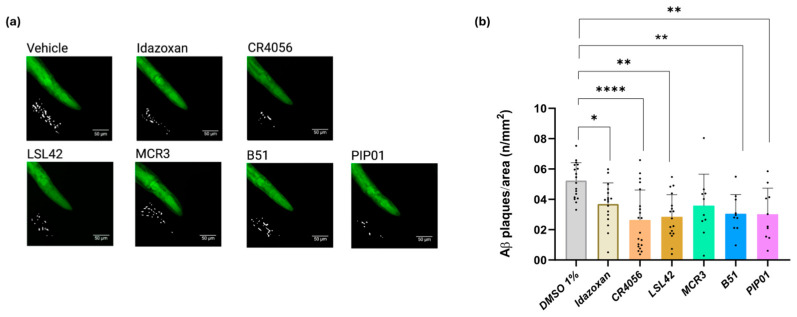
(**a**) Representative images of Amyloid-β aggregates measured in *C. elegans* (CL2006) treated with Vehicle, Idazoxan, CR4056, LSL42, MCR3, B51, and PIP01 at 0.5 μM. All images have a representative inset of the quantifying method using ImageJ. (**b**) Quantification of positive Aβ aggregates in the head region of the *C. elegans* CL2006 strain treated with Idazoxan, CR4056, LSL42, MCR3, B51, and PIP01. Values are represented as mean ± SEM (*n* = 3, with 25 worms in each group for each experiment); * *p* < 0.05; ** *p* < 0.01; **** *p* < 0.0001 vs. vehicle.

We further investigated the effects of chronic treatment with selected I2-IR ligands on Aβ aggregation, a key hallmark of AD, using thioflavin-S (ThS) staining in the transgenic CL2006 strain [38]. Idazoxan and CR4056 significantly reduced Aβ deposition (Figure 4a,b). Most of the other I2-IR ligands also exhibited a similar trend, although the degree of reduction varied statistically (Table A2). Specifically, LSL42, B51, and PIP01 produced a statistically significant decrease in the number of Aβ aggregates, whereas MCR3 reduced aggregates but did not reach statistical significance (Figure 4a,b).

### 2.5. I2-IR Ligands Modulate sod-1 Gene Expression in CL2006

**Figure 5:**
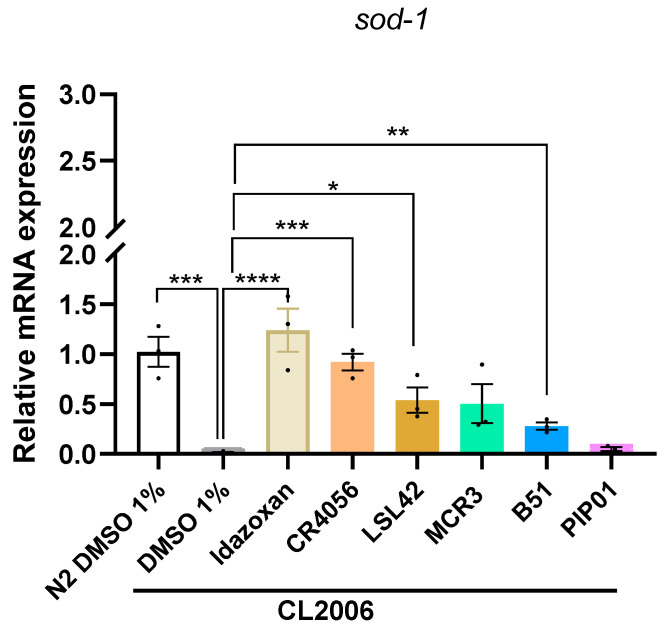
Representative gene expression of sod-1 in *C. elegans* (Bristol N2 and CL2006) treated with Vehicle, Idazoxan, CR4056, LSL42, MCR3, B51 and PIP01. Gene expression levels were determined by real-time PCR. The data corresponds to the mean ± SEM; *n* = 3, with 500–700 worms in each group for each experiment. One-Way ANOVA; statistically significant vs. CL2006; * *p* < 0.05; ** *p* < 0.01; *** *p* < 0.001; **** *p* < 0.0001.

To further investigate the potential effects of I2-IR ligands in OS, gene expression of *sod-1* was evaluated for the compounds selected based on a neuroprotective test performed in advance or on published results in mice [21]: Idazoxan, CR4056, MCR3, LSL42, B51, and PIP01. Consistent with the OS tolerance results, most tested compounds significantly increased *sod-1* expression in the CL2006 strain after 0.5 μM Idazoxan, CR4056, LSL42, and B51 compared to the vehicle group (Figure 5). However, the magnitude of *sod-1* upregulation by B51 was notably lower compared to other I2-ligands, which may explain its limited protective effect in the oxidative stress survival assay (Figure 3). In contrast, PIP01 did not show statistically significant upregulation of *sod-1* expression despite its effects in other assays, suggesting that its neuroprotective mechanisms may involve alternative pathways independent of SOD-1 modulation. Further mechanistic studies are required to elucidate the concrete pathways engaged by B51 and PIP01.

### 2.6. I2-IR Ligands Reduced Htt-513 Aggregates in the EAK-103 C. elegans Strain

**Figure 6:**
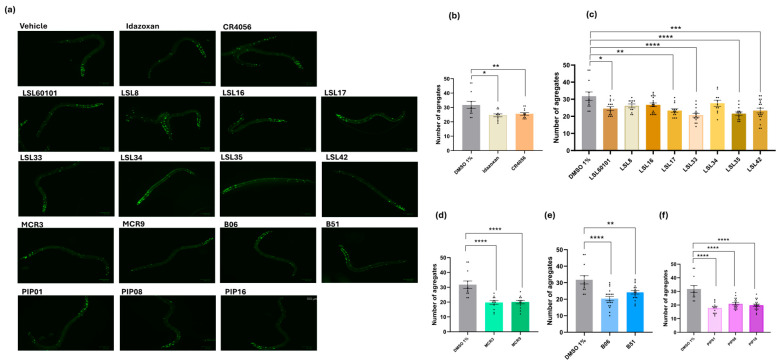
(**a**) Representative fluorescent micrographs of *C. elegans* (EAK103) treated with I2-IR ligands at day 1 of adulthood and Number of huntingtin 513 (Htt513) aggregates in the head of *C. elegans* (EAK103) treated with (**b**) Idazoxan, CR4056; (**c**) LSL60101, LSL8, LSL16, LSL17, LSL33, LSL34, LSL35, LSL42; (**d**) MCR3, MCR9; (**e**) B06, B51 and (**f**) PIP01, PIP08 and PIP16 at 0.5 µM. The data corresponds to the mean ± SEM; *n* = 3, with 15–20 worms in each group for each experiment. One-Way ANOVA; * *p* < 0.05; ** *p* < 0.01; *** *p* < 0.001; **** *p* < 0.0001 vs. vehicle.

HD pathology has previously been studied in *C. elegans* strains, making this model a feasible tool for studying pharmacological interventions to slow disease progression. To assess the effectiveness of I2-IR ligands in HD, we measured the aggregation of Htt-513 in the EAK-103 strain, which is associated with the onset of symptoms [39]. Surprisingly, most LSLs, MCRs, and PIPs families of I2-IR ligands, including Idazoxan and CR4056, significantly reduced Htt-513 aggregation in EAK103 compared to the vehicle group (Figure 6). These results showed, for the first time, a putative beneficial effect of I2-IR ligands reducing huntingtin aggregates in an in vivo model, and led us to propose the I2-IRs as a therapeutic target for HD.

### 2.7. I2-IR Ligands Reduces sod-1 Gene Expression in EAK103 Strain

**Figure 7:**
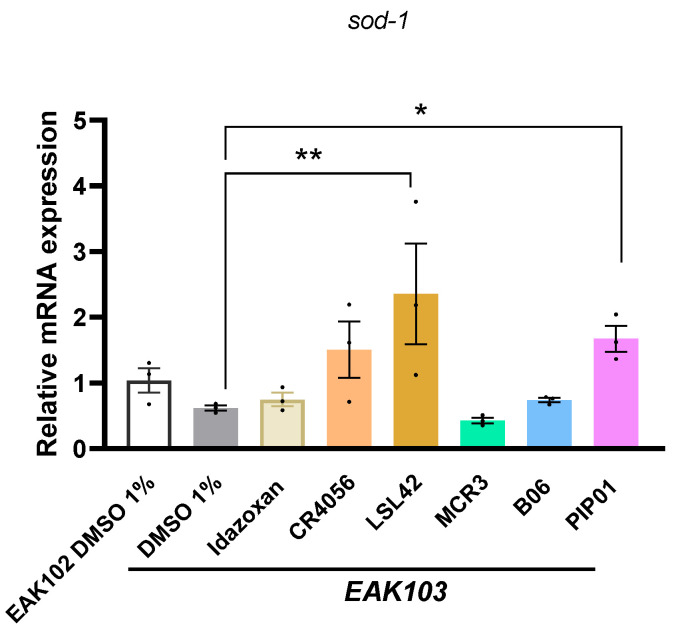
Quantification of *sod-1* gene expression in *C. elegans* (EAK102 and EAK103) treated with I2-IR ligands. Gene expression levels were determined by real-time PCR. Data are presented as mean ± SEM; *n* = 3, with 500–700 worms per group for each experiment. One-Way ANOVA; statistically significant vs. EAK103; * *p* < 0.05; ** *p* < 0.01.

To establish the mechanism of action for I2-IR ligands in the EAK103 strain, we studied their potential antioxidant role by measuring *sod-1* gene expression. Although the results did not show significant differences in this gene between the control strain (EAK-102) and EAK-103, important changes in gene expression were observed after I2-IR ligand treatment. Regarding the expression of *sod-1* in the EAK103 strain (HD), we found a significant increase in the expression of *sod-1* with LSL42 and PIP01 but not with Idazoxan, CR4056, MCR3, or B06, suggesting that the mechanism involved in the observed effect would be different between those I2-IR ligands related to chemical structure (Figure 6 and Figure 7). Further studies centered on HD are required to elucidate the beneficial effects of I2-IR modulation in this model.

## 3. Discussion

Neurodegenerative diseases impose substantial medical and public health burdens on populations throughout the world. AD is one of the major neurodegenerative diseases, which is expected to rise dramatically with the increase in life expectancy in many countries [1,4,5]. AD is characterized by progressive neuronal loss associated with Amyloid-β aggregation and tau pathology [7,40], leading to cognitive decline, dementia, and other neuropsychiatric symptoms such as anxiety, apathy, and irritability, which could appear with the progression of the disease [41]. Current AD treatments primarily provide symptomatic relief through cholinesterase inhibitors and memantine, while recent advances include monoclonal antibodies such as lecanemab and donanemab, which modestly slow early cognitive decline by targeting amyloid plaques. Despite over two decades of research focusing on amyloid and tau proteins, clinical success rates remain low, and therapies offer limited benefits in significantly altering disease progression [7,8,42].

Research shows that I2-IR ligands reduced inflammation by inhibiting microglial activation and decreasing the production of pro-inflammatory cytokines [17], supporting their potential role in neurodegenerative diseases, such as AD or HD. In this way, the search for effective pharmacological approaches for AD and HD is an ongoing field of research, which we address here by proposing *C. elegans* as a screening tool. Transgenic *C. elegans* expressing human Aβ has been a useful tool in AD research, considering its short life span and its ability to develop muscle-associated deposits reactive to amyloid-specific dyes and the concomitant progressive paralysis phenotype [30]. Regarding the advantages of the transgenic *C. elegans* models of AD, the CL2006 strain was employed to assess the effect of I2-IR ligands, for the first time, by the BINs family and B06 [22].

Following this first evidence in *C. elegans* regarding the beneficial effects of I2-IR ligands mentioned above, here we studied new families of I2-IR ligands, with robust evidence of neuroprotection in mouse models (see affinities for I2-IR in human tissues and relevant in vivo data in Table A1) [20,21,27], including well-established I2-IR compounds such as Idazoxan (preclinical pharmacological standard) and CR4056 (clinically evaluated for pain) in the CL2006 strain. This approach aimed to validate *C. elegans* as a screening tool for novel I2-IR ligands. Importantly, the aim of this work was to evaluate the neuroprotective potential of mammalian I_2_-IR ligands in *C. elegans* models of AD and HD, and to validate these models as a screening platform. Accordingly, our findings support the use of a phenotypic tool that enables the study of I_2_-IR-related pathway modulation, while definitive receptor identification and genetic characterization will be pursued in more complex in vivo systems, such as mouse models or human tissues. Firstly, we established that chronic treatment in the *C. elegans* N2 (Bristol) strain was safe within a concentration range between 0.1 and 10 μM, compared to the vehicle-treated (DMSO 1%) group. Only the highest dose (10 μM) of CR4056 reduced worm viability. Moreover, the lack of toxicity agrees with published data obtained in SH-SY5Y cells for most of the I2-IR ligands tested in this study [22,23,24,26].

Since OS is a common feature of neurodegenerative diseases such as AD and HD, the antioxidant potential of I2-IR ligands was evaluated in the *C. elegans* N2 strain. The results aligned with toxicity assays, suggesting antioxidant effects across multiple ligand families, which is consistent with potential modulation, based on prior reports and the observed phenotypic outcomes in animal models for compounds such as MCR5, LSL60101, B06, and PIP01 (see affinities for I2-IR in human tissues and relevant in vivo data in Table A1) [16,20,21,25,26]. These neuroprotective effects were corroborated in cell and mouse models, where I2-IR ligands reduced oxidative damage by modulating antioxidant enzymes (Superoxide dismutase 1, SOD-1; Glutathione peroxidase 1, GPX-1; or Aldehyde Oxidase 1, AOX1) and prooxidant markers (Heme Oxygenase 1, HMOX-1 or Cyclooxygenase 2, COX-2). Treatments with MCR5, MCR9, LSL60101, B06, and BU224 altered OS markers and activating pathways such as nuclear factor erythroid 2-related factor 2 (Nfr-2), demonstrating a strong correlation between in vivo and in vitro findings and supporting the therapeutic potential of I2-IR ligands in neurodegeneration. For example, the neuroprotective role of I2-IR ligands was demonstrated in SH-5Y-SY cells challenged with 6-hydroxydopamine (6-OHDA) as a cellular model of Parkinson’s disease, which induces OS in human dopaminergic cells or in HT-22 hippocampal mouse neuronal cells treated with glutamate, as an excitotoxic stimulus mimicking AD [22,23,26]. In those studies, and others that included I2-IR ligands, it showed that the I2-IR antioxidant protective effect can be related to the expression and activity of detoxifying enzymes (SOD-1, GPX-1, and AOX1), important key enzymes in cellular antioxidant-defense, or prooxidant ones, such as HMOX-1, COX-2. Concretely, some of these markers also significantly increased in AD mouse models after treatment with MCR5, MCR9, LSL60101, or B06 [20,21,25,27,43]. Additionally, other OS markers, such as *Aox1* or *Cox2*, and the activation of nuclear factor erythroid 2-related factor 2 (Nrf-2) were found to be modified after BU224 treatment [17].

It is known that AD patients’ brains have a significant extent of oxidative damage associated with the abnormal marked accumulation of Aβ and the deposition of neurofibrillary tangles [44,45,46]. As we mentioned, the Aβ aggregation, tau pathology, synaptic and neuron loss are characteristic hallmarks of AD. Accordingly, with this relationship, I2-IR ligand families were tested in an AD model of *C. elegans*, the CL2006 strain, characterized by a loss in muscular movements because of amyloid deposits in muscle cells. The I2-IR ligands Idazoxan and CR4056 increased statistically significantly the swimming movements, then reduced paralysis. Other chemical families showed an improvement in comparison to CL2006, which does not reach significance, but indicates a beneficial effect, as shown in mouse models (see affinities for I2-IR in human tissues and relevant in vivo data in Table A1). Therefore, we assessed the effect of the selected I2-IR ligands based on previous results in Aβ aggregation that, as mentioned, is related to the progressive paralysis observed in CL2006, employing the ThS staining. Results demonstrated the reduction in Aβ aggregates in worms treated with I2-IR ligands, corroborating the beneficial effects observed in paralysis assays. These results are in agreement with those reported with I2-IR ligands in mice models of AD, where a neuroprotection related to Aβ aggregates reduction was observed (see affinities for I2-IR in human tissues and relevant in vivo data in Table A1) [20,25].

In consistency with the OS tolerance results, we analyzed the mRNA expression of *sod-1*. SOD protects cells from excess reactive oxygen species (ROS) by converting them into less reactive hydrogen peroxide. It is suggested that SODs can play a protective role in neurodegeneration [47]. In *C. elegans*, *sod-1* is the ortholog of SOD-1, a superoxide dismutase, responsible for down-regulation of ROS and preventing oxidative DNA damage to cells [48,49,50]. On the one hand, a significant increase in the expression of *sod-1* in most of the I2-IR ligand-treated worms was observed, suggesting that the effect observed in the OS tolerance assay could be related to the increase in detoxifying activity of SOD-1, and on the other hand, mirrored results obtained in mouse models [51]. In contrast, although B51 induced a modest increase in *sod-1* expression, this was not reflected in the OS tolerance assay, likely because the upregulation was smaller than that observed for Idazoxan or CR4056. This divergent profile indicates that sod-1 mRNA levels are not sufficient to predict survival in the OS assay for this scaffold and suggests that B51 may engage additional, potentially pro-oxidant or off-target pathways that override its limited antioxidant signaling. Accordingly, B51 should be considered a lower-priority candidate pending more detailed mechanistic and toxicity studies. Further studies to address the mechanism of action involved should be done for B51, which has not been passed on to animal models yet.

Current treatments for HD focus mostly on alleviating symptoms but lack in modifying the progression of the disease; in that matter, I2-IR ligands emerge as promising candidates to face HD pathology [18]. A characteristic hallmark of HD is the aberrant accumulation of mutant huntingtin protein, resulting in mitochondrial dysfunction, oxidative stress, neuronal dysfunction, and progressive neuronal degeneration [52,53]. There is also evidence that HD is typified by neuronal loss, intracellular inclusions, astrogliosis, and microglial activation [54]. In HD, it is demonstrated that I2-IR density is decreased in the post-mortem patient’s brain [11,55], but its relevance has not been completely elucidated. Therefore, the results in OS tolerance proved that I2-IR ligands, such as Idazoxan, will be very promising therapeutic candidates for HD. Several transgenic *C. elegans* strains express human mHTT fragments with polyglutamine (polyQ) expansions that are a useful approach to understanding protein aggregation, cellular stress responses, and potential therapeutic pathways, specifically the EAK-103 strain, developed by Lee [34], expresses a fragment of mutant human huntingtin protein (Htt513 with 128 glutamines), allowing the study of paralysis phenotype and Htt aggregates under control or intervention conditions. The aberrant huntingtin protein (Htt-513) was evaluated in this study, in the EAK103 strain, compared with the EAK102 (wild-type) strain, where we could see the difference in Htt-513 aggregates, demonstrating that the model is accurate to assess the effect of Htt-513. As seen in the results, I2-IR ligands, including Idazoxan and CR4056, were effective in reducing the number of Htt-513 aggregates. Previous studies demonstrate that preformed polyQ aggregates are highly toxic when directed to the cell nucleus; thus, pharmacological intervention may focus its attention on inhibiting toxic aggregate formation, as it has been shown to have beneficial effects in a mouse model of HD [4,56]. Therefore, our results with I2-IR ligands prove that they could be considered as a possible therapeutic approach for HD. Likewise, changes in locomotion and aggregate burden could, in principle, be influenced by developmental or technical factors rather than by neuroprotection alone. However, because we used synchronized populations, standardized conditions, and multiple complementary readouts, a purely artifactual explanation cannot be excluded but appears less likely.

Protein carbonyls, markers of oxidative stress, appear to be increased in HD brains, indicating that ROS are overproduced [50,57,58,59]. As mentioned, the overexpression of ROS could be regulated via SOD1; to establish a body of evidence supporting I2-IR in HD, we analyzed the mRNA expression of *sod-1* (homolog of human SOD-1) in EAK103 after treatment with I2-IR ligands. The results showed that only LSL42 and PIP01 upregulated *sod-1* expression. This dissociation between *sod-1* expression and the anti-aggregation effects was observed for most ligands and indicates that SOD-1-independent mechanisms—likely involving autophagy, proteasomal activity, or broader proteostasis networks—may drive the neuroprotective effects in this HD model. Importantly, changes in *sod-1* mRNA expression alone are not sufficient to conclude activation of the full antioxidant pathway, particularly in the absence of direct ROS measurements, protein-level validation of SOD-1, or functional rescue data. These findings are therefore presented as preliminary mechanistic indicators that warrant further biochemical investigation. In contrast, *sod-1* upregulation correlates with Aβ reduction in the AD model, suggesting disease-specific roles of this gene across different neurodegenerative contexts. In contrast, *sod-1* upregulation correlates with Aβ reduction in the AD model, suggesting disease-specific roles.

Our results reinforce the therapeutic potential of I_2_-IR modulation in neurodegenerative diseases such as AD and HD, highlighting selective ligands as promising candidates for further development. Notably, this work provides the first in vivo evidence that I_2_-IR ligands can attenuate neurodegeneration in the *C. elegans* HD model EAK103, although additional studies are required to elucidate the molecular mechanisms underlying these effects. These findings, together with the outcomes obtained in the CL2006 AD model, support the use of *C. elegans* as an efficient early-stage platform for screening I_2_-IR ligands prior to advancing to mammalian systems such as mice (see affinities for I2-IR in human tissues and relevant in vivo data in Table A1).

## 4. Materials and Methods

### 4.1. C. elegans Strains and Maintenance

The following *C. elegans* strains were used in this study: N2, a wild-type reference strain; CL2006, a transgenic strain expressing human Aβ peptide 1-42; EAK102 and EAK103, transgenic strains expressing Huntingtin protein with 15 or 128 glutamine repeats, respectively. All strains were obtained from the Caenorhabditis Genetics Center (CGC). The worms were cultivated using standard protocols [60]. N2 and worms were grown at 20 °C, while CL2006, EAK102, and EAK103 were maintained at 16 °C in temperature-regulated incubators. The nematodes were raised on solid nematode growth medium (NGM) plates, with *E. coli* strain OP50 serving as their food source. Nematodes were synchronized by alkaline hypochlorite treatment to obtain in each experimental well: 25–30 L1 stage nematodes suspended in S-medium, the compounds being evaluated at their respective concentrations, and inactivated OP50 dissolved in S-medium complete. *E. coli* OP50 bacteria had been inactivated through freeze–thaw cycles. The bacterial density was adjusted to achieve an optical density (OD595) between 0.7 and 0.9, as measured using a microplate reader, before the experiment was initiated.

### 4.2. Experimental Design

The main objective of this study was to evaluate the neuroprotective effects of I_2_-imidazoline receptor (I_2_-IR) ligands, including Idazoxan hydrochloride (CAS# 79944-56-2, Sigma, St. Louis, MO, USA), CR4056, LSL60101, LSL8, LSL16, LSL17, LSL33, LSL34, LSL35, LSL42, MCR3, MCR9, B06, B51, PIP01, PIP08, and PIP16. For primary screening, compounds were tested at 0.1–10 μM, a concentration range selected based on prior in vitro and in vivo data for I_2_-IR ligands indicating robust neuroprotective effects with limited toxicity. A 4-day exposure window was chosen to encompass the critical period during which Aβ and Htt-513 aggregates and the associated behavioral phenotypes emerge in the CL2006 and EAK103 strains. The first phase assessed the effects of the selected compounds in *C. elegans* using an automated movement assay, and the lowest dose that produced consistent improvement (0.5 μM) was then used in secondary assays, including oxidative stress tolerance, Aβ and Huntingtin 513 aggregation, and Sod-1 evaluation, all relevant to AD- and HD-like pathology.

**Figure 8:**
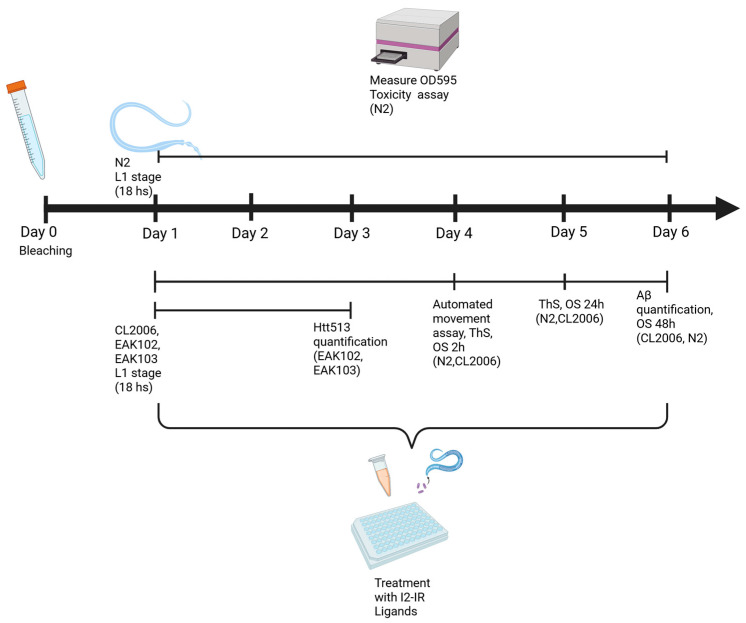
Workflow of standard treatment with I2-IR. Created in BioRender. Bellver Sanchis, A. (2026) https://BioRender.com/6w87qdp (accessed on 26 March 2026).

Test substances were initially solubilized in pure DMSO and diluted with Milli-Q purified water to achieve final well concentrations between 0.1 and 10 μM, with 1% DMSO in all conditions. A systematic, parallel screening approach was used to evaluate 18 I_2_-IR ligands from five chemical families, with all compounds tested together within the same biological replicates (*n* = 3 per assay) and standardized vehicle controls (1% DMSO) included in every replicate to minimize batch variability. The results are presented in separate figure panels arranged by chemical family to enhance visual clarity and facilitate structure–activity relationship analysis, while the shared control values across figures reflect the use of synchronized experimental batches rather than data duplication; Figure 8 summarizes the standard worm treatment protocol.

### 4.3. Chemical Synthesis of the Tested Compounds

**Figure 9:**
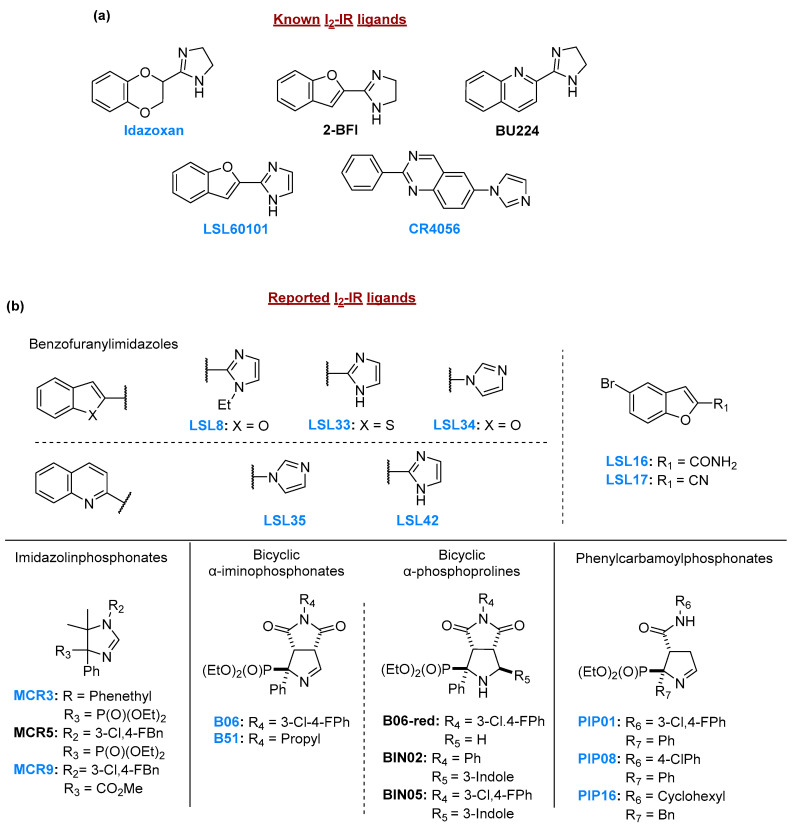
Chemical structures of compounds referred to, and the names of the compounds tested, are highlighted in blue. (**a**) Known I2-IR ligands embodying an imidazoline/imidazole heterocyclic nucleus. (**b**) Structurally diverse I2-IR ligands previously reported by our group.

Idazoxan was purchased from commercial sources. The rest of the compounds tested were synthesized following experimental procedures previously reported in the literature. The most relevant data are detailed below, along with the corresponding bibliographic references. CR4056 and LSL60101 were prepared after a few synthetic steps [24,61]. MCR5/MCR3/MCR9 were obtained after a multicomponent reaction was performed in a microwave oven [62]. Compounds with bicyclic iminophosphonate structure, B06 and B51, resulted from the diastereoselective [3+2] cycloaddition reaction of diethyl isocianomethylphosphonate or α-substituted derivatives and diversely substituted maleimides [25]. The opening in basic conditions of the compounds mentioned above led to PIP01, PIP08, and PIP16 with a (3-phenylcarbamoyl-3,4-dihydro-2*H*-pyrrol-2-yl)phosphonate nucleus [26]. The nucleophilic addition of indole to the imine functional group of members of the family of bicyclic iminophosphonates leads to access to BIN02 and BIN05, and the reduction in the imine gives access to B06-red [22]. The benzofuranyl imidazoles were synthesized from their corresponding heteroderivatives. Specifically, compounds LSL8, LSL33, and LSL42 were obtained from the cyano derivative. Compounds LSL34 and LSL35 were synthesized from the bromo derivative, while LSL16 and LSL17 were derived from the carboxylic acid derivative [23,24]. All compounds tested are >95% pure by HPLC and are fully characterized (IR, ^1^H, and ^13^C NMR) in the cited references. Figure 9 shows the chemical structures of the compounds studied.

### 4.4. C. elegans Methodology

#### 4.4.1. Food Clearance Assay

N2 (wild-type) worms underwent a liquid-format drug assay. The worms were cultured at 20 °C with continuous shaking at 180 rpm for 5 days. Each well contained a total volume of 60 μL, including 25–30 L1-stage larvae diluted in S-medium, I2-IR ligands at the designated concentrations, and freeze–thaw inactivated OP50 bacteria suspended in S-medium complete solution to a final OD595 of 0.8, as measured with a microplate reader. The tested concentrations of I2-IR ligands ranged from 0.1 μM to 10 μM. Control wells included 1% DMSO (vehicle) and 5% DMSO (toxic condition), while blank wells contained either S-medium alone or S-medium complete, without eggs or OP50, respectively. The impact of the compounds on *C. elegans* physiology was assessed by measuring the rate of OP50 suspension consumption, which serves as an indicator of growth, survival, or fecundity. OD595 readings were taken daily. Of note, preliminary toxicity screening employed N2 at 20 °C following standard *C. elegans* protocols [60]. Working doses (0.1–1 μM) were selected with a 10-fold safety margin below the established toxicity threshold (10 μM CR4056). Strain-specific toxicity profiling at different temperatures represents an important direction for future comprehensive safety characterization. The assays were performed in triplicate (*n* = 3), with at least 120 animals tested per compound concentration.

#### 4.4.2. Automated Movement Assay

After 4 days of chronic treatment in liquid culture with the different I_2_-imidazoline receptor ligands, both *C. elegans* N2 and CL2006 strains were transferred to the WMicroTracker™ Mini system (PhylumTech, Santa Fe, Argentina) to quantify locomotor activity. Movement was automatically recorded over a 20 min period, and the assay was performed in triplicate (*n* = 3) with a total of 120 worms analyzed per compound.

#### 4.4.3. Oxidative Tolerance Assay

To conduct this assay, we selected a dose of 0.5 μM, which had previously shown improvement in the movement assay. Adult wild-type worms (N2) were treated for 4 days at 20 °C and then transferred to plates containing 6.2 mM t-butyl hydroperoxide (CAS# 75-91-2, Alfa Aesar, Kandel, Germany) in NGM agar. The worms were incubated on these plates at 20 °C for 2 h. Following incubation, the worms were moved to fresh NGM plates seeded with OP50 bacteria and devoid of t-butyl hydroperoxide. Observations were conducted 2-, 24-, and 48 h post-intervention, with worms scored dead if they failed to respond to repeated prodding with a pick. This oxidative stress survival assay with t-butyl hydroperoxide was performed according to standard *C. elegans* survival protocols [63]. As a positive control, 58 µM vitamin C (L-(+)-Ascorbic acid 99%, CAS# 50-81-7, Alfa Aesar, Kandel, Germany) was used.

### 4.5. Thioflavin-S Staining Aβ Aggregation

Adult CL2006 *C. elegans* were treated with the selected dose for 4 days, then fixed in 4% paraformaldehyde (PBS, pH 7.5) for 24 h at 4 °C. Worms were permeabilized in 5% β-mercaptoethanol, 1% Triton X-100, and 125 mM Tris (pH 7.5) at 37 °C for 24 h, then stained with 0.125% Thioflavin S (ThS; Sigma, CAS 1326-12-1) in 50% ethanol for 2 min, destained for 2 min, and washed with PBS. Samples (~10 μL) were mounted in Fluoromount G (Electron Microscopy Sciences, Hatfield, PA, USA, Cat.#17984-25) and imaged using a Leica Thunder fluorescence microscope (40× objective).

Fluorescence images were processed in ImageJ2-FIJI (version 2.16/1.54p). Each worm head region was defined, converted to greyscale, and segmented using a Triangle threshold. Background was corrected (rolling ball radius = 10 px), smoothed (Median filter, 1 px), and re-thresholded by the Otsu method. Binary images were refined with Open and Watershed functions to isolate Aβ plaques. Plaques were quantified and normalized to head area (plaque density). Approximately 25 worms per condition were analyzed across three independent experiments (~75 animals/group), providing sufficient statistical power to detect changes in Aβ aggregation.

### 4.6. Quantification of Huntingtin-513 Aggregate Number

Htt-513 aggregate quantification was conducted on the EAK102 and EAK103 strains on day 1 of adulthood. For the evaluation of Htt-513 aggregate number, worms were exposed to treatment in liquid culture for 3 days at 20 °C. For fluorescence analysis of Htt513 (Q128) in live animals, day 1 adults were immobilized using 0.4% sodium azide, mounted on 2% agarose pads, and covered with a coverslip. Fluorescent micrographs were captured using a ZOE GREEN microscope (BioRad, Barcelona, Spain) and the aggregate count in each image was determined by counting Htt513-positive spots with ImageJ2-FIJI (version 2.16/1.54p). The results were expressed as the number of aggregates per image. Aggregate quantification was performed on 15–20 worms per treatment condition per independent replicate (*n* = 3 independent experiments), for a total of approximately 45–60 animals analyzed per treatment group. This sample size was determined based on the robustness of the Htt513-GFP fluorescent signal and the consistency of aggregate counts across biological replicates, ensuring adequate statistical power for detecting treatment effects [34].

### 4.7. RNA Extraction and Gene Determination

The total RNA was isolated from treated *C. elegans* samples using TRIzol^®^ reagent following the manufacturer’s protocol (Bioline Reagents, London, UK). RNA yield, purity, and quality were assessed using a NanoDrop™ ND-1000 spectrophotometer (Thermo Fisher, Waltham, MA, USA).

For reverse transcription, 2 μg of mRNA was converted to cDNA using a high-capacity cDNA reverse transcription kit (Applied Biosystems, Foster City, CA, USA). Real-time PCR was performed using SYBR^®^ Green chemistry on a StepOnePlus Detection System (Applied Biosystems, Foster City, CA, USA). The reaction mixture contained 6.75 μL of cDNA (2 μg), 0.75 μL of each primer (100 nM), and 6.75 μL of SYBR Green PCR Master Mix (2×) (Applied Biosystems) (Table A3). Data analysis was conducted using the comparative cycle threshold (ΔΔCt) method, with a housekeeping gene used to normalize variations in sample loading and preparation.

### 4.8. Statistical Analysis

Data are expressed as mean ± SEM from three independent biological replicates. For survival and locomotion assays, each replicate included 120–150 worms per group, whereas imaging-based endpoints and gene expression analyses were performed on 15–25 and 500–700 worms per group, respectively. Data distribution was assessed for approximate normality, and one-way ANOVA was applied when this assumption was reasonably met. Post hoc multiple-comparison tests (Tukey’s) were used to compare individual treatment groups, with a significance level of *p* < 0.05. All analyses were performed using GraphPad Prism v9.0.

## 5. Conclusions

The advantages of *C. elegans*, including its rapid life cycle, genetic tractability, low maintenance cost, and compatibility with high-throughput in vivo assays, make it particularly suitable for early drug discovery, where time, resources, and compound throughput can be limiting. Its strong conservation of molecular pathways relevant to human neurodegeneration further enhances the translational value of the hits identified during our screening cascade. Importantly, *C. elegans* remains more versatile than many other simple in vivo models, as it allows simultaneous evaluation of toxicity and diverse biological activities (e.g., antioxidant and neuroprotective), a feature we leveraged in our research.

Our study also strengthens the growing body of evidence demonstrating the feasibility of *C. elegans* for modeling complex neurodegenerative phenotypes. By expressing human disease-associated proteins such as Aβ, Tau, or mutant huntingtin, the worm recapitulates key pathological features of AD, PD, ALS, and HD. Its optical transparency enables direct in vivo visualization of neuronal degeneration, protein aggregation, and progression of pathology, critical advantages for mechanistic studies and pharmacological validation.

In essence, while mammalian models remain essential for late-stage preclinical research, the speed, cost-effectiveness, throughput capacity, and genetic accessibility of *C. elegans* position it as a powerful initial screening tool. By bridging the gap between molecular-level investigations and resource-intensive vertebrate experiments, this model organism significantly enhances the efficiency and feasibility of early neurotherapeutic discovery efforts, in our case, AD and HD.

## Data Availability

The original contributions presented in this study are included in the article. Further inquiries can be directed to the corresponding authors.

## Abbreviations

The following abbreviations are used in this manuscript:

Aβ	Amyloid-β
AD	Alzheimer’s Disease
Aox-1	Aldehyde Oxidase 1
BBB	Blood–Brain Barrier
cDNA	Complementary DNA
Cox-2	Cycloxygenase
DMSO	Dimethyl sulfoxide
GFAP	Glial fibrillary acidic protein
GPX	Glutathion peroxydase
HD	Huntington’s Disease
Hmox-1	Heme Oxygenase 1
Ht	Ht aggregation
Htt513	Huntingtin 513
I2-IR	Imidazoline I2 receptor
NGM	Nematode growth medium
NRF2	Nuclear factor erythroid 2-related factor 2
OD595	Optical density OD595
OS	Oxidative stress
PBS	Phosphate-buffered saline
PCR	Polymerase Chain Reaction
polyQ	polyglutamine
ROS	Reactive oxygen species
SAMP8	Senescence-accelerated mouse prone 8
SEM	Standard error of the mean
SOD-1	Superoxide dismutase 1
ThS	Thioflavin-S

## Appendix A

**Table A1:** I2-IR ligands pharmacological characteristics (human tissues) and main effects found in animals’ models of AD.

Compounds	[^3^H]-2-BFI, I_2_ One Site (p*K*_i_) ^a^ I_2_ One Site Two Sites H/L; (p*K*_iH_/p*K*_iL_) High Affinity Site %	[^3^H]-RX821002 α_2_ (p*K*_i_)	I_2_/α_2_ Selectivity ^b^	Effects in AD Mice Models ^d^	References
**Idazoxan**	7.41 ± 0.63	8.35 ± 0.16	-		[25]
**CR4056**	7.72 ± 0.31/5.45 ± 0.15 29 ± 6	2.65 ± 1.24	117,490		[25]
**2-BFI**	9.87 ± 0.33/7.94 ± 0.11 21 ± 5	6.64 ± 0.38	1698		[25]
**BU224**	8.67 ^c^	4.92 ^c^	5623		[13]
**LSL60101**	6.67 ± 0.09 8.17 ± 0.19/6.02 ± 0.10 34 ± 4	3.18 ± 0.17	3090	BC; βA/pTau; OS; NI	[24,28,54]
**LSL8**	5.05 ± 0.10 7.09 ± 0.42/4.72 ± 0.15 22 ± 6	3.77 ± 0.08	19		[24]
**LSL33**	5.95 ± 0.24 10.1 ± 0.57/5.68 ± 0.25 27 ± 6	4.70 ± 0.12	-	BC; NP; NI; NT	[23]
**LSL34**	5.58 ± 0.31 9.23 ± 0.36/4.64 ± 0.32 37 ± 6	5.40 ± 0.21	-		[23]
**LSL35**	5.49 ± 0.14	5.24 ± 0.26	-		[23]
**LSL42**	5.24 ± 0.12	5.32 ± 0.24	-		[23]
**MCR3**	8.19 ± 0.27	4.18 ± 0.09	10,233	BC; βA/pTau; NP; OS; NI	[16]
**MCR5**	9.42 ± 0.16	6.76 ± 0.22	457		[16,21,46]
**MCR9**	8.85 ± 0.21	5.58 ± 0.14	1862		[16,21]
**B06**	8.56 ± 0.32 8.61 ± 0.28/4.29 ± 0.20 37 ± 4	6.27 ± 0.56	195	BC; βA/pTau; OS; NI	[25,46]
**B51**	4.02 ± 0.41	±	-		[25]
**BIN02**	7.31 ± 0.39	3.61 ± 0.32	5012		[22]
**BIN05**	3.89 ± 0.19 8.18 ± 0.43/3.56 ± 0.22 21 ± 3	5.51 ± 0.24	-		[22]
**PIP01**	9.98 ± 0.74	9.43 ± 0.22	3	BC; OS; NI; NT	[26]
**PIP08**	<3	<3	-		[26]
**PIP16**	5.22 ± 0.14	7.97 ± 0.36	-		[26]

^a^ The best fit of the data for CR4056, LSL60101, LSL8, LSL33, LSL34 and B06 was to a two-site binding model of binding with high p*K*_i_ (p*K*_iH_) and low p*K*_i_ (p*K*_iL_) affinities for both binding sites respectively. ^b^ Selectivity I_2_-IRs/α_2_-ARs expressed as the antilog (p*K*_i_ I_2_-IRs-p*K*_i_ α_2_-ARs). ^c^ Results from brain rat membranes. ^d^ Beneficial effect found in mice: Behavior and cognitive (BG); AD Hallmarks (βA/pTau); Neuroplasticity (NP); Oxidative stress (OS); Neuroinflammation (NI); Neurotrophins (NT).

**Table A2:** Results from oxidative stress and ThS staining. Results presented as Mean ± SEM.

I2-IR Compound	% of Survival (Oxidative Stress)			Aβ Plaques/Area (ThS Staining)
	2 h	24 h	48 h	
**Vehicle**	100.00 ± 0.00	*	25.20 ± 6.44	*
**Idazoxan 0.5 μM**	100.00 ± 0.00	*	70.90 ± 14.65	*
**CR4056 0.5 μM**	91.67 ± 8.33	*	38.33 ± 13.09	*
**LSL60101 0.5 μM**	100.00 ± 0.00	56.67 ± 13.47	4.44 ± 2.93	0.38 ± 0.04
**LSL8 0.5 μM**	90.00 ± 10.00	17.41 ± 6.80	2.96 ± 2.93	0.40 ± 0.05
**LSL16 0.5 μM**	100.00 ± 0.00	73.21 ± 21.90	11.48 ± 4.82	0.44 ± 0.03
**LSL17 0.5 μM**	100.00 ± 0.00	31.11 ± 13.09	8.89 ± 8.89	0.39 ± 0.04
**LSL33 0.5 μM**	100.00 ± 0.00	51.11 ± 6.76	17.50 ± 7.98	0.37 ± 0.05
**LSL34 0.5 μM**	19.17 ± 14.17	1.04 ± 1.39	0.00 ± 0.00	0.40 ± 0.03
**LSL35 0.5 μM**	100.00 ± 0.00	60.00 ± 8.82	26.67 ± 5.78	0.34 ± 0.03
**LSL42 0.5 μM**	97.41 ± 2.58	*	76.67 ± 16.67	*
**MCR3 0.5 μM**	100.00 ± 0.00	*	35.56 ± 13.09	*
**MCR9 0.5 μM**	97.38 ± 1.70	35.16 ± 1.51	27.78 ± 1.93	0.36 ± 0.04
**B06 0.5 μM**	97.11 ± 2.88	65.56 ± 17.35	41.11 ± 12.81	0.47 ± 0.04
**B51 0.5 μM**	97.12 ± 2.89	*	4.62 ± 1.03	*
**PIP01 0.5 μM**	100.00 ± 0.00	*	10.11 ± 5.78	*
**PIP08 0.5 μM**	100.00 ± 0.00	51.11 ± 17.89	7.78 ± 6.18	0.38 ± 0.03
**PIP16 0.5 μM**	95.00 ± 3.95	16.67 ± 8.39	3.33 ± 2.89	0.32 ± 0.05

* Results presented in Figure 3 and Figure 4.

**Table A3:** Primers used in qPCR studies.

	Forward Primer (5′-3′)	Reverse Primer (3′-5′)
*act-1*	ATCACCGCTCTTGCCCCATC	GGCCGGACTCGTCGTATTCTTG
*sod-1*	TGTCGTTGGCCGATCTATGG	GTGGAGATTCAGAGACGCGA
